# Extracellular matrix modulates the spatial hepatic features in hepatocyte-like cells derived from human embryonic stem cells

**DOI:** 10.1186/s13287-023-03542-x

**Published:** 2023-11-01

**Authors:** Faiza Farhan, Manjari Trivedi, Priscilla Di Wu, Wei Cui

**Affiliations:** Institute of Reproductive and Developmental Biology, Faculty of Medicine, Imperial College London, Hammersmith Hospital Campus, Du Cane Road, London, W12 0NN UK

**Keywords:** Extracellular matrix (ECM), Hepatocyte, hESC differentiation, Hepatic zonation, Matrigel, Collagen

## Abstract

**Background:**

Human pluripotent stem cell (hPSC)-derived hepatocyte-like cells (HLCs) can provide a valuable in vitro model for disease modelling and drug development. However, generating HLCs with characteristics comparable to hepatocytes in vivo is challenging. Extracellular matrix (ECM) plays an important role in supporting liver development and hepatocyte functions, but their impact on hepatocyte differentiation and maturation during hPSC differentiation remains unclear. Here, we investigate the effects of two ECM components—Matrigel and type I collagen on hepatic differentiation of human embryonic stem cells (hESCs).

**Methods:**

hESC-derived HLCs were generated through multistage differentiation in two-dimensional (2D) and three-dimensional (3D) cultures, incorporating either type I collagen or Matrigel during hepatic specification and maturation. The resulting HLCs was characterized for their gene expression and functionality using various molecular and cellular techniques.

**Results:**

Our results showed that HLCs cultured with collagen exhibited a significant increase in albumin and alpha-1 anti-trypsin expression with reduced AFP compared to HLCs cultured with Matrigel. They also secreted more urea than Matrigel cultures. However, these HLCs exhibited lower CYP3A4 activity and glycogen storage than those cultured with Matrigel. These functional differences in HLCs between collagen and Matrigel cultures closely resembled the hepatocytes of periportal and pericentral zones, respectively.

**Conclusion:**

Our study demonstrates that Matrigel and collagen have differential effects on the differentiation and functionality of HLCs, which resemble, to an extent, hepatic zonation in the liver lobules. Our finding has an important impact on the generation of hPSC-HLCs for biomedical and medical applications.

**Supplementary Information:**

The online version contains supplementary material available at 10.1186/s13287-023-03542-x.

## Introduction

The liver is an important organ in our body as it plays an essential role in metabolism and detoxification and is responsible for maintaining body homeostasis [1]. Therefore, it is not surprising that liver disease is one of the major contributors to human morbidity and mortality [2]. Furthermore, the liver is also associated with the pathogenesis of diseases, for example malaria, in which parasites require liver cells to amplify themselves before causing the disease [3]. Developing new treatments or prevention strategies for liver and liver-associated diseases will greatly reduce the burden on healthcare services and improve human health. Thus, it is necessary to understand the mechanisms of human liver pathogenesis, and establishing both in vitro and in vivo liver models can greatly facilitate such studies.

Since hepatocytes constitute over 70% of the liver mass and carry out most of the liver functions, obtaining functional hepatocytes is vital for developing an effective liver model [1]. However, the availability of primary human hepatocytes from donors is limited, and hepatocarcinoma cell lines are inadequate models for non-cancer studies. Therefore, human pluripotent stem cell (hPSC)-derived hepatocytes offer a consistent and reliable source for human hepatocytes as hPSCs can self-renew indefinitely and are capable of producing hepatocytes on demand [4–6]. The differentiation procedure from hPSCs to hepatocytes emulates the embryonic development of the liver, in which the hepatocytes originate from the definitive endodermal epithelium of the embryonic foregut and then become the hepatic endoderm influenced by the signals from the adjacent mesodermal cells [7]. The hepatic endoderm gives rise to non-polarized hepatoblasts that invade adjacent mesenchyme to generate the liver bud. The hepatoblasts can then differentiate into either hepatocytes or biliary epithelial cells (BECs) depending on the signalling regulation [8–11]. Although the current approaches can efficiently generate hepatocyte-like cells (HLCs) from hPSCs, the maturation and functions of these HLCs are still not yet comparable to primary hepatocytes [3, 12–16].

Hepatocyte maturation is a complex process, requiring interaction between cells, extracellular matrix (ECM) and various substances secreted by other cell types, such as Kupffer cells, endothelial cells, and stellate cells [17–19]. Furthermore, the liver is a structurally complex and highly organized organ composed of repeating hexagonally shaped functional lobules with a portal triad in each corner and a central vein in the centre (Additional file 1: Fig. S1A). Blood enters the lobules from the portal triad and flows radially towards a draining central vein, which divides the intralobular hepatocytes into three distinct functional zones, with zone 1 closest to the portal triad and zone 3 around the central vein [20]. Increasing evidence shows that the functionality of a hepatocyte is also regulated by spatial signals relevant to its position in the liver lobules [21–23]. As yet, it is a huge challenge to generate functional hepatocytes and liver tissues from hPSCs.

ECM plays an important role in liver development and hepatocyte differentiation through cell–ECM interactions, so evidently, aberrant ECM expression can lead to liver disorders [24, 25]. The liver contains multiple ECM components with collagens and fibronectin as the main constituents, while laminin is predominantly found in the bile ductules [26]. However, the effects of these ECM components in the modulation of hepatocyte maturation and functions are not completely understood. In this study, we investigated the effects of growth factor-reduced Matrigel (abbreviated as MG hereafter) and type I collagen (as COL hereafter) on the differentiation, maturation, and function of human embryonic stem cell-derived HLCs in both monolayer and 3D spheroid culture systems. We show that MG and COL have differential effects on the functions of the HLCs and generate HLCs with functional features of distinct hepatic zones 3 and 1, respectively.

## Methods

### hESCs culture and hepatic differentiation

H1 and H7 hESC lines, purchased from WiCell Research Institute (Madison, WI, http://www.wicell.org), were routinely cultured on MG-coated plates in mouse embryonic fibroblast-conditioned knockout serum replacement (KSR) medium supplemented with 10 ng/ml of bFGF (brief as MEF-CM) as previously described [27]. All the cells were cultured in incubators at 37 °C, 5% CO_2_.

**Fig. 1:**
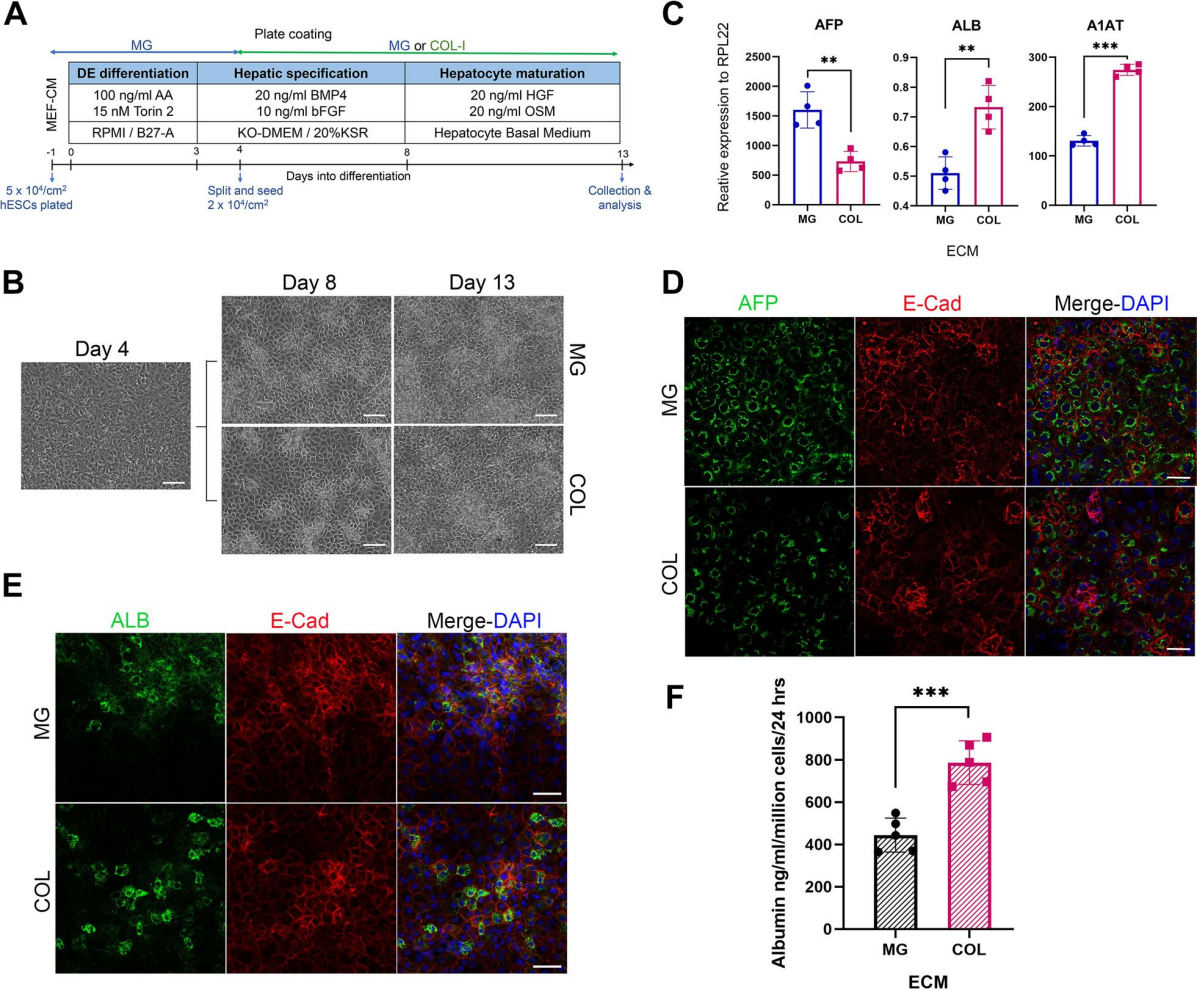
Matrigel and collagen differentially affect the production of hepatic proteins in hESC-derived HLCs in monolayer cultures. **A** Schematic depicting the differentiation procedure. AA, Activin A; bFGF: basic fibroblast growth factor; BMP4, bone morphogenesis protein 4; HGF, hepatocyte growth factor; KSR: knockout serum replacement; and OSM, oncostatin M. **B** Representative phase-contrast images of cells at indicated differentiation days from H1 hESCs. Scale bar = 100 µm. **C** mRNA expression of indicated genes by RT-qPCR in H1 HLCs at day 13 of the differentiation. Data are presented as mean ± SD of independent differentiation experiments (*n* = 4). **D, E** Representative images of immunostaining with indicated antibodies in H1 HLCs. Scale bar = 50 µm. **F** Albumin secretion from H1 HLCs cultured in Matrigel (MG) or collagen-I (COL). Data presented as mean ± SD of independent differentiation experiments (*n* = 5). *, **, and *** represent *p* ≤ 0.05, 0.005, and 0.0005, respectively, by unpaired two-tailed *t*-test

Hepatic differentiation (Fig. 1A) was modified from the previously published method [28] and initiated by plating accutase-dissociated 5 × 10^5^ hESCs onto 6-well plates pre-coated with MG (Corning) in MEF-CM medium. The next day, the medium was changed to RPMI-B27 supplemented with 100-ng/ml Activin A (Qkine) and 15-nM Torin2 (Bio-Techne) for 3 days, then the medium was replaced with KSR medium supplemented with 20-ng/ml BMP4 and 10-ng/ml bFGF. On day 4, the cells were dissociated by trypsin into single cells, plated at 2 × 10^4^/cm^2^ onto MG- (0.3 mg/ml) or COL (1 mg/ml)-coated plate, and continued to culture for 4 more days in the same medium. Finally, the maturation medium, hepatocyte culture medium (Lonza) supplemented with 20-ng/ml oncostatin M (OSM) (Biotech) and 20-ng/ml hepatocyte growth factor (HGF), was applied for 5 days. During this process, all the cells were fed with fresh medium daily. All growth factors were purchased from PeproTech unless stated otherwise.

**Fig. 2:**
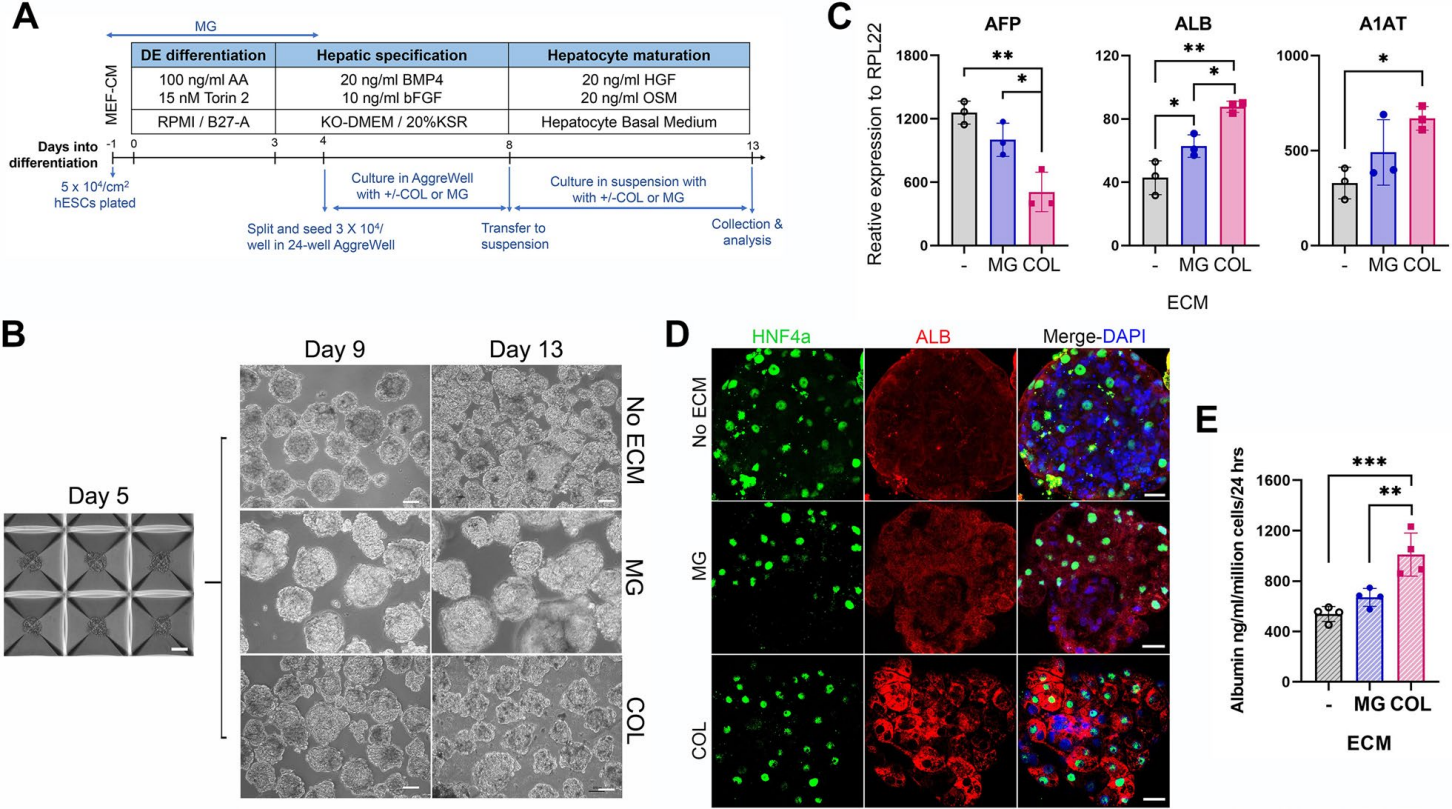
ECM affects the production of hepatic proteins in hESC-derived HLCs in 3D cultures. **A** Schematic illustrating the experimental procedure. **B** Representative phase-contrast images of cultures at indicated differentiation days with indicated ECM. Scale bar = 100 µm. **C** mRNA expression of indicated genes by RT-qPCR in HLCs at day 13 of the differentiation. Data are presented as mean ± SD of independent differentiation experiments (*n* = 3). **D** Representative images of immunostaining with indicated antibodies in HLC spheroids. Scale bar = 25 µm. **E** Albumin secretion from HLCs cultured with indicated ECM. Data presented as mean ± SD of independent differentiation experiments (*n* = 4). *, **, and *** represent *P* < 0.05, 0.005, and 0.0005, respectively, by one-way ANOVA. ECM: extracellular matrix

The 3D spheroid cultures were performed using 24-well AggreWell™ 400 plates (STEMCELL Technologies) following the manufacturer’s instructions with modification. Briefly, following pre-treating the plate with Anti-Adherence Rinsing Solution (STEMCELL Technologies), the day 4 hepatic differentiating cells were dissociated with trypsin and plated into the AggreWell™ plate at 3 × 10^4^/well in KSR medium containing either MG (0.3 mg/ml) or COL (0.03 µg/ml) as well as BMP4 and bFGF as shown in Fig. 2A. The plate was immediately centrifuged at 150 × *g* for 10 min, and cells were cultured for 48 h before half of the medium was refreshed on day 6. On day 8, the spheroids were transferred to 35-mm bacteria culture dishes and cultured in suspension in 5-ml hepatic maturation medium containing the same ECM for 5 days with half-medium change every alternate day.

### Reverse transcription–quantitative PCR (RT–qPCR)

Total RNA was isolated from cells using the Tri Reagent (Sigma), and reverse transcription was performed with Protoscript II Reverse Transcriptase (NEB). PCR was carried out using SYBR Green JumpStar Taq ReadyMix (Merck) in the StepOnePlus real-time PCR system (Applied Biosystems). Primer sequences are provided in Additional file 1: Table S1, and two housekeeping genes, β-ACTIN and RPL22, were used, which showed similar patterns. Thus, the relative quantification of target gene expression was calculated using the 2^−ΔCt^ method with RPL22 as the normalizer.

### Immunostaining

Cells were fixed with 4% paraformaldehyde for 15 min, followed by incubation for 1 h with PBS containing 10% donkey serum, 3% BSA, and 0.3% triton (Merck) for permeabilization and blocking non-specific binding. The cells were then incubated overnight with primary antibodies at 4 °C, followed by with fluorescence-conjugated secondary antibodies for 1 h, with PBS washes in between. Finally, the cells were stained with DAPI (Merck) for nuclei and mounted using Mowiol 4–88 solution. All procedures were performed at room temperature unless stated otherwise. Antibodies used: mouse anti-albumin (Merck, A6684, 1:100), rabbit anti-AFP (Abcam, ab169552, 1:500), rabbit anti-E-cadherin (CST, #3195, 1:200), rabbit anti-glucokinase (Abcam, ab88056, 1:100), rabbit anti-LEF1 (CST, #2230, 1:200), donkey anti-rabbit IgG (H + L), Alexa Fluor™ 488 (Fisher, A21206, 1:500), donkey anti-rabbit IgG (H + L), and Alexa Fluor® 568 conjugate (Fisher, A10042, 1:500).

### Periodic acid–Schiff (PAS) staining

Following 4% paraformaldehyde fixation and PBS washes, cells were stained using the PAS kit following the manufacturer’s instructions (Merck). Briefly, cells were incubated for 5 min in 1% periodic acid solution, followed by washing with distilled water for several minutes and then immersing into Schiff’s reagent for 20 min. After washing with tap water, cells were counterstained with haematoxylin solution for 20 s before visualizing them under the Nikon TE2000 microscope.

### Albumin ELISA, urea assay, and CYP3A4 activity assay

Supernatants of the HLC cultures were collected at day 13 of the differentiation after 24-h incubation with cells, and the secreted ALB protein levels were measured by the human Albumin ELISA kit (Merck) following the manufacturer’s instructions. Medium incubated for 24 h in the absence of cells was used as a blank control. After collecting supernatants, cells were counted. Also, the urea assay was performed similarly using the urea assay kit (Abcam) following the instructions of the manufacturer. CYP3A4 activities were measured at the end of differentiation (day 13) in either untreated or after rifampicin (25 μM) stimulation using the P450-Glo CYP3A4 assay (luciferin-IPA) kit (Promega) following the manufacturer’s instructions.

### BODIPY staining

About 4% paraformaldehyde-fixed HLCs were incubated in the dark with BODIPY 493/503 (Fisher, 1:1000 in PBS) for 30 min and then counterstained with DAPI after washing. The LD signals were finally captured using a Leica SP5 II confocal fluorescent microscope. Quantification and image analysis were performed using CellProfiler software (https://cellprofiler.org/).

### Statistical analysis

Unpaired two-tailed *t*-test, Welch *t*-test, and one-way or two-way ANOVA were used for statistical analysis with GraphPad Prism 8 or 9 (GraphPad, USA) on at least three independent experimental samples. *P* < 0.05 was considered a statistically significant difference.

## Results

### COL improved the albumin secretion of hESC-derived HLCs

COL is one of the most abundant ECMs in the liver, while MG is the most commonly used ECM for hESC culture and hepatocyte differentiation [12, 13, 29]. MG is a solubilized basement membrane preparation extracted from the Engelbreth–Holm–Swarm (EHS) mouse sarcoma and is primarily composed of laminin, collagen IV, and heparan sulphate proteoglycan [30]. Given the distinct composition of MG and COL, we asked whether different components of the ECM may contribute differently to the maturation and function of hESC-derived HLCs. To address this, early hepatic differentiating cells that were generated following the definitive endoderm formation from hESCs (both H1 and H7 lines) were split and plated onto either MG- or COL-coated plates and continued to culture for hepatic specification and maturation (Fig. 1A). MG was used at the same concentration (0.3 mg/ml) as it was for hESC culture, while COL was initially applied at various concentrations ranging from 0.03 to 2 mg/ml. The differentiating hepatic cells did not attach well to the plates when < 1 mg/ml COL was applied; thus, 1 mg/ml was finally used for coating the plate. During the differentiation process, cells from both MG and COL cultures showed no clear difference in their overall morphology, although COL cells looked slightly bigger than the MG cells (Fig. 1B; Additional file 1: Fig. S1B). However, the expression of key hepatocyte markers, albumin (ALB), alpha-1 anti-trypsin (A1AT), and alpha-fetoprotein (AFP), showed significant differences between the two cultures (Fig. 1C–E; Additional file 1: Fig. S1C). The transcripts of ALB and A1AT were significantly more abundant in HLCs on COL-coated dishes than on MG-coated ones, whereas AFP mRNA was higher in MG HLCs (Fig. 1C; Additional file 1: Fig. S1C). The protein expression of ALB and AFP corresponded to the transcript expression, showing higher ALB and lower AFP in HLCs cultured on COL than MG (Fig. 1D and E; Additional file 1: Fig. S1D). Moreover, albumin secretion was higher in COL HLCs than in MG (Fig. 1F; Additional file 1: Fig. S1E). Since adult hepatocytes produce more ALB while foetal hepatocytes express higher AFP [31], the data suggest that COL may enhance the maturation of hESC-derived HLCs.

To further validate this finding, we also examined the effects of MG and COL on HLCs in 3D cultures. hESCs were initially differentiated into early hepatic cells as in 2D culture, which were then collected on day 4 of the differentiation (Fig. 2A) and plated into 24-well AggreWell plates in hepatic specification medium supplemented with MG or COL for further differentiation and maturation until day 13 (Fig. 2A and B). The MG was used at the same concentration as in 2D culture as it has demonstrated efficacy in Hep3B spheroids [32]. However, when COL was used at the concentrations of 1 mg/ml and 0.3 mg/ml for spheroid culture, it formed clumps. Consequently, a concentration of 0.03 µg/ml COL was selected for spheroid culture after optimization. The resulting spheroids were analysed for the expression of AFP, ALB, and A1AT. Like the 2D cultures, higher expression of ALB and A1AT as well as higher albumin secretion were detected in spheroids of COL cultures than that of MG, although supplementing MG exhibited improved ALB and A1AT expression compared to no ECM (Fig. 2C–E; Additional file 1: Fig. S2A and B). These data further support that COL enhances the maturity of the HLCs. Moreover, they also revealed lower AFP and higher ALB expression in 3D cultures than in 2D cultures regardless of the ECM (Fig. 1 vs. Fig. 2), indicative of a more efficient hepatocyte maturation in 3D cultures.

### COL and MG differentially affect ureagenesis and CYP3A4 activity in hESC-HLCs

**Fig. 3:**
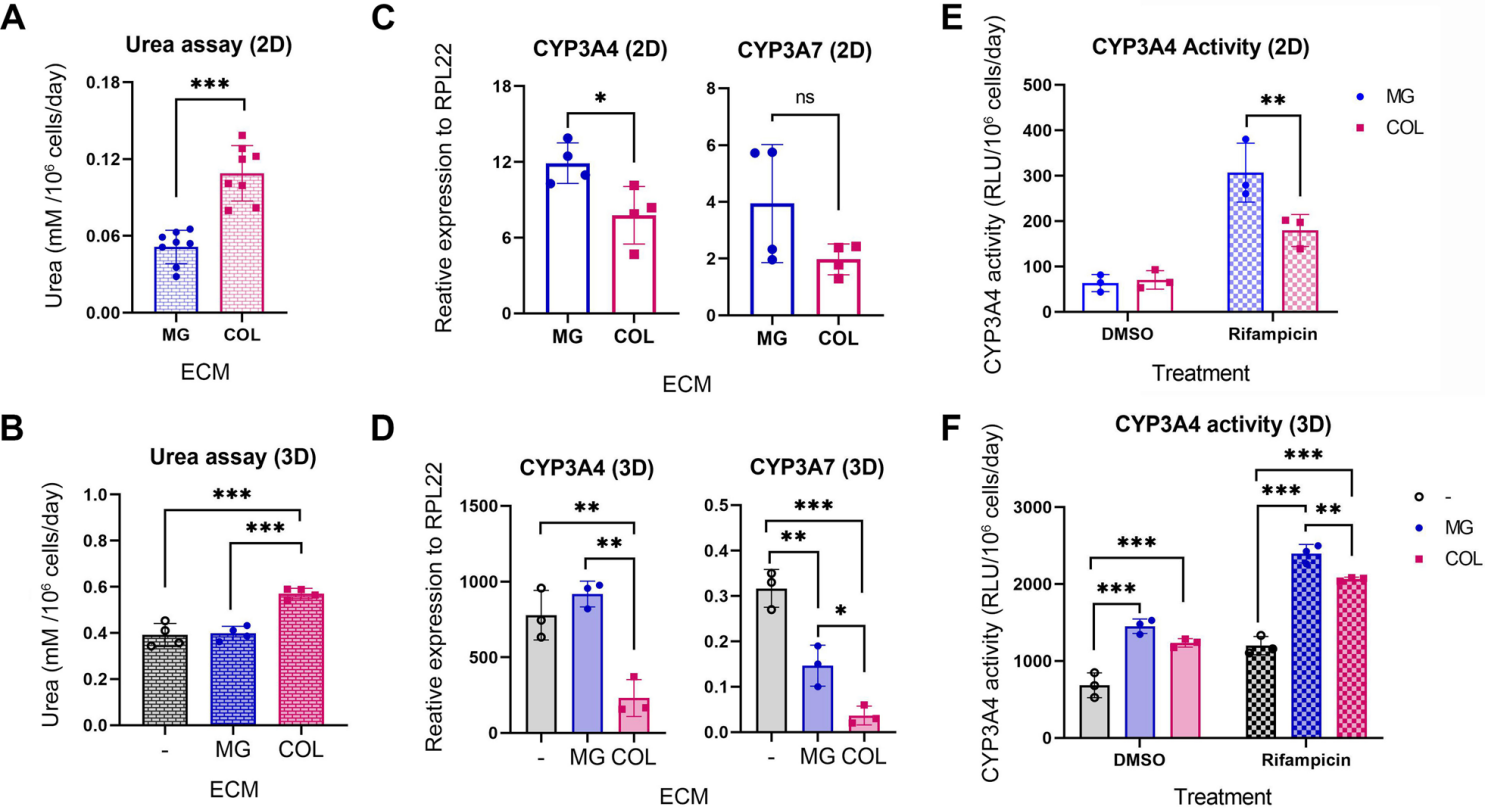
COL and MG have differential effects on ureagenesis and CYP3A expression. **A, B** Urea assay in H1 HLCs in 2D (**A**) or 3D (**B**) cultures with indicated ECM. Data are presented as mean ± SD from independent differentiation experiments in 2D (*n* = 8) and 3D (*n* = 4) systems. **C, D** mRNA expression of CYP3A4 and CYP3A7 in H1 HLCs in 2D (**C**, *n* = 4) or 3D (**D**, *n* = 3) culture systems with indicated ECM by RT-qPCR. **E, F** CYP3A4 activities in H1 HLCs in 2D (**E**, *n* = 3) or 3D (**F**, *n* = 3) cultures with indicated ECM in the presence or absence of rifampicin stimulation. RLU, relative light unit. *, **, and *** represent *P* < 0.05, 0.005, and 0.0005, respectively, by unpaired two-tailed *t*-test (**A**, **C**) and one-way (**B**, **D**) or two-way (**E**, **F**) ANOVA

The liver is an important organ for detoxification; thus, to further explore the impact of ECM on these functions in hESC-derived HLCs, we compared ureagenesis in HLCs cultured with COL and MG using both 2D and 3D culture systems. In both 2D and 3D cultures, HLCs cultured with COL produced more urea than those cultured with MG (Fig. 3A and B; Additional file 1: Fig. S3A and B). Interestingly, MG did not show an apparent effect on ureagenesis in the 3D culture compared to cells grown without any additional ECM (Fig. 3A and B; Additional file 1: Fig. S3A and B). Moreover, ureagenesis was over tenfold higher in 3D cultures than in 2D regardless of the ECM supplementation. Overall, these results support our observations in Figs. 1 and 2 that both COL and 3D culturing improve the maturation and functionality of hESC-derived HLCs.

Next, the expression of cytochrome P450 enzymes, particularly CYP3A4 and CYP3A7, was examined in these cells. CYP3A4 is the most abundant drug-metabolizing enzyme in the human liver, responsible for the phase I metabolism of dietary compounds and over 50% of prescribed drugs, while CYP3A7 is the predominant P450 enzyme in human foetal and infant liver tissues [33, 34]. Surprisingly, both CYP3A4 and CYP3A7 transcripts were higher in the HLCs cultured with MG than those with COL, regardless of whether they were grown in 2D or 3D cultures (Fig. 2C and D; Additional file 1: Fig. S3C and D). Although there was no difference in the basal activity of CYP3A4 in HLCs between MG and COL cultures, a significantly higher CYP3A4 activity was detected in the HLCs of MG cultures than those of COL upon rifampicin stimulation, despite all of them showing an overall increase in response to rifampicin (Fig. 3E and F; Additional file 1: Fig. S3E and F). Together, these data suggest that HLC maturation and function might be differentially regulated by COL and MG and cannot be simply described as positive effects of either COL or MG on HLC culture.

### Higher glycogen accumulation in HLCs cultured with MG than with COL

**Fig. 4:**
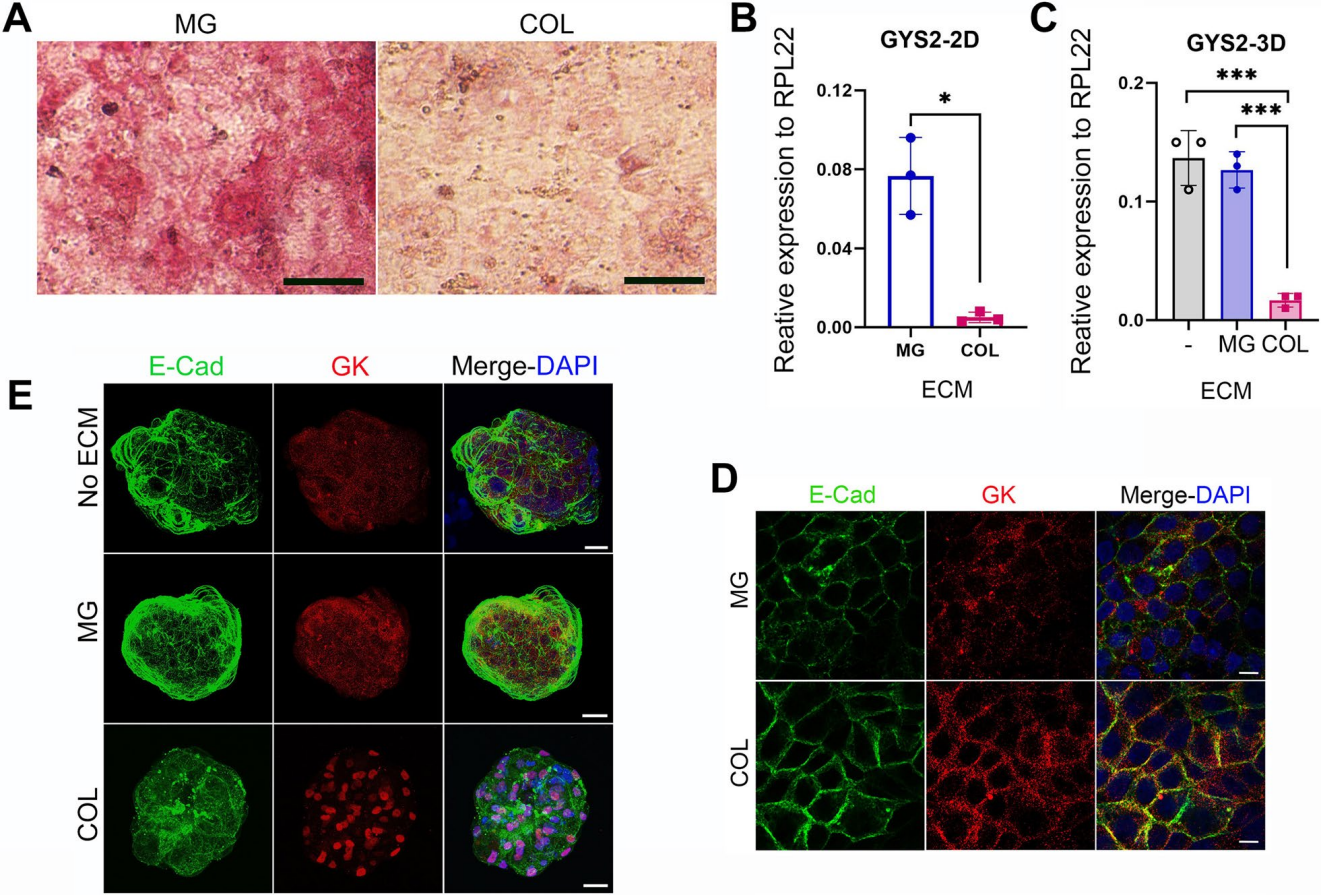
COL and MG have different effects on glucose metabolism in hESC-derived HLCs in both 2D and 3D cultures. **A** Glycogen storage in 2D-cultured HLCs on Matrigel (MG) or type I collagen (COL) by PAS staining. Scale bar = 50 µm. **B, C** RT-qPCR showing GYS2 mRNA expression in HLCs cultured with indicated ECM in 2D (B) or 3D (C) cultures. Data are presented as mean ± SD of three independent differentiation experiments (*n* = 3). * and *** represent *P* < 0.05 and 0.0005, respectively, by either Welch’s *t*-test (**B**) or one-way ANOVA (**C**). **D** Immunostaining with indicated antibodies in 2D-cultured HLCs on either MG or COL. Scale bar = 10 µm. **E** Maximum intensity projection of similar staining as in D on 3D-cultured HLCs with indicated ECM. Scale bar = 25 µm

Since MG and COL revealed diverse effects on different functions of hESC-derived HLCs, we asked whether they may also have differential effects on cell metabolism. Glucose and lipid metabolism are important functions of hepatocytes. The conversion of glucose into glycogen by the liver is a major pathway to maintain blood glucose homeostasis. Therefore, we studied glycogen synthesis and storage in the HLCs cultured with MG or COL. Interestingly, the HLCs cultured on MG showed stronger PAS (periodic acid–Schiff) staining than cells grown on COL (Fig. 4A; Additional file 1: Fig. S4A). Moreover, liver-specific glycogen synthase (GYS2) also exhibited higher expression in MG-cultured HLCs than COL HLCs (Fig. 4B and C; Additional file 1: Fig S4B and C). Together, these data indicate that HLCs cultured on MG have more robust glucose–glycogen conversion than those cultured on COL. Additionally, we also looked at glucokinase (GK) expression in our cultures because it plays a key role in glycogen synthesis and glycolysis by catalyzing glucose conversion to glucose-6-phosphate and is tightly regulated, only being active when it is in the cytoplasm [35]. In our 2D cultures, we detected GK in the cytoplasm of HLCs on both COL and MG (Fig. 4D); whereas, in 3D cultures, GK showed distinct localization depending on whether the cells were cultured with COL or MG (Fig. 4E). It exhibited a strong nuclear expression in COL 3D cultures whereas revealed more diffuse, predominantly cytoplasmic localization in MG cultures (Fig. 4E). These data suggest that MG may promote more glycogen synthesis and glycolysis in HLCs than COL, particularly in the 3D culture system.

### Distinct lipid droplet patterns in HLCs cultured on MG and COL

**Fig. 5:**
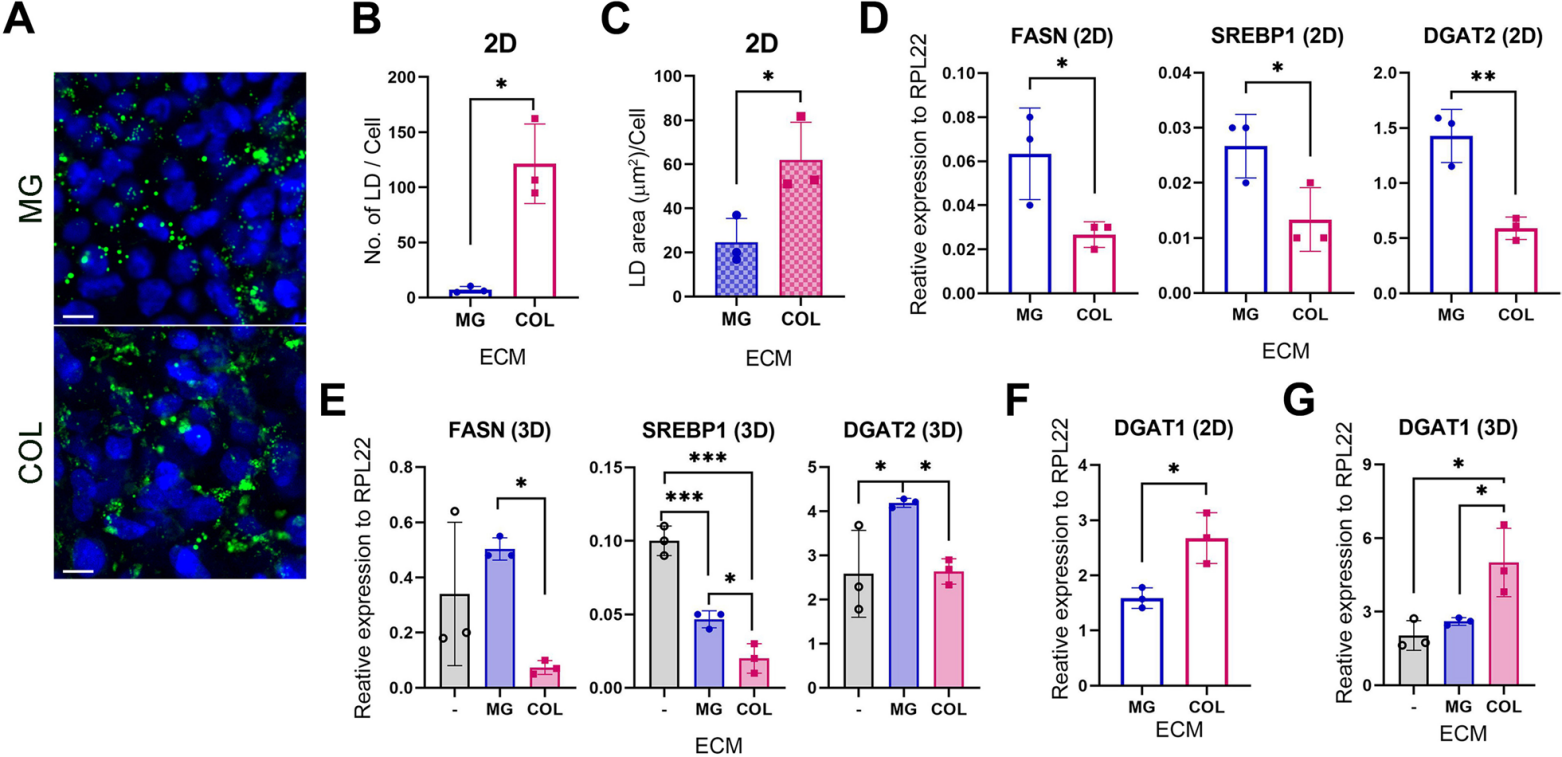
MG and COL differentially affect the size of lipid droplets (LD) in hESC-derived HLCs. **A** BODIPY staining of LDs in H1 HLCs cultured on MG- or COL-coated plates. Scale bar = 10 µm. **B, C** Bar graphs showing the number (**B**) and the size distribution (**C**) of LDs in HLCs. **D-G** RT-qPCR analysis on the expression of indicated genes involved in LD formation in H1 hESC-derived HLCs with indicated ECM in both 2D (**D**, **F**) and 3D (**E**, **G**) cultures. *, **, and *** represent *P* < 0.05, 0.005, and 0.0005, respectively, by either Welch’s t-test (**B**), unpaired two-tailed *t*-test (**C**, **D**, **F**), or one-way ANOVA (**E**, **G**) with three independent biological samples for each condition (*n* = 3 for all)

Next, we examined the effects of MG and COL on lipid metabolism by studying the presence and size of lipid droplets (LDs) because hepatocytes store both dietary fatty acids and de novo-generated lipids in the form of LDs [36]. BODIPY staining, a method that stains LDs, was applied to HLCs on day 13 of their differentiation on MG or COL. Both MG and COL HLCs exhibited positive staining of LDs (Fig. 5A; Additional file 1: Fig. S5A), and their quantitation showed that HLCs cultured on COL had significantly more and larger LDs than HLCs cultured on MG (Fig. 5B and C). However, the genes involved in lipogenesis, such as sterol regulatory element-binding protein 1 (SREBP1), its downstream lipogenic enzyme fatty acid synthase (FASN), and the fatty acid esterification enzyme DGAT2 (diacyl glycerol transferase-2), were expressed significantly higher in MG-cultured HLCs than in COL HLCs (Fig. 5D and E; Additional file 1: Fig. S5B and C), indicating a lower lipogenesis in the COL HLCs. These data suggest that higher LD content in COL-cultured HLCs may not result from increased lipogenesis but possibly from high fatty acid (FA) uptake from the medium. It has been reported previously that COL increases triglyceride content and free FA uptake in human podocytes by activating discoidin domain receptor 1 (DDR1) and consequently increasing FA uptake [37, 38]. Excessive FA is esterified to LD through the endoplasmic reticulum (ER) enzyme, DGAT1, to prevent lipotoxicity [39, 40]. Indeed, when we checked DGAT1 expression in our cultures, HLCs on COL expressed significantly higher DGAT1 than on MG (Fig. 5F and G; Additional file 1: Fig. S5D and E), supporting that higher LDs in COL-cultured HLCs might be associated with an increased FA uptake.

### MG and COL differentially modulate HLC maturation and functionality representing discrete hepatic zonal features

**Fig. 6:**
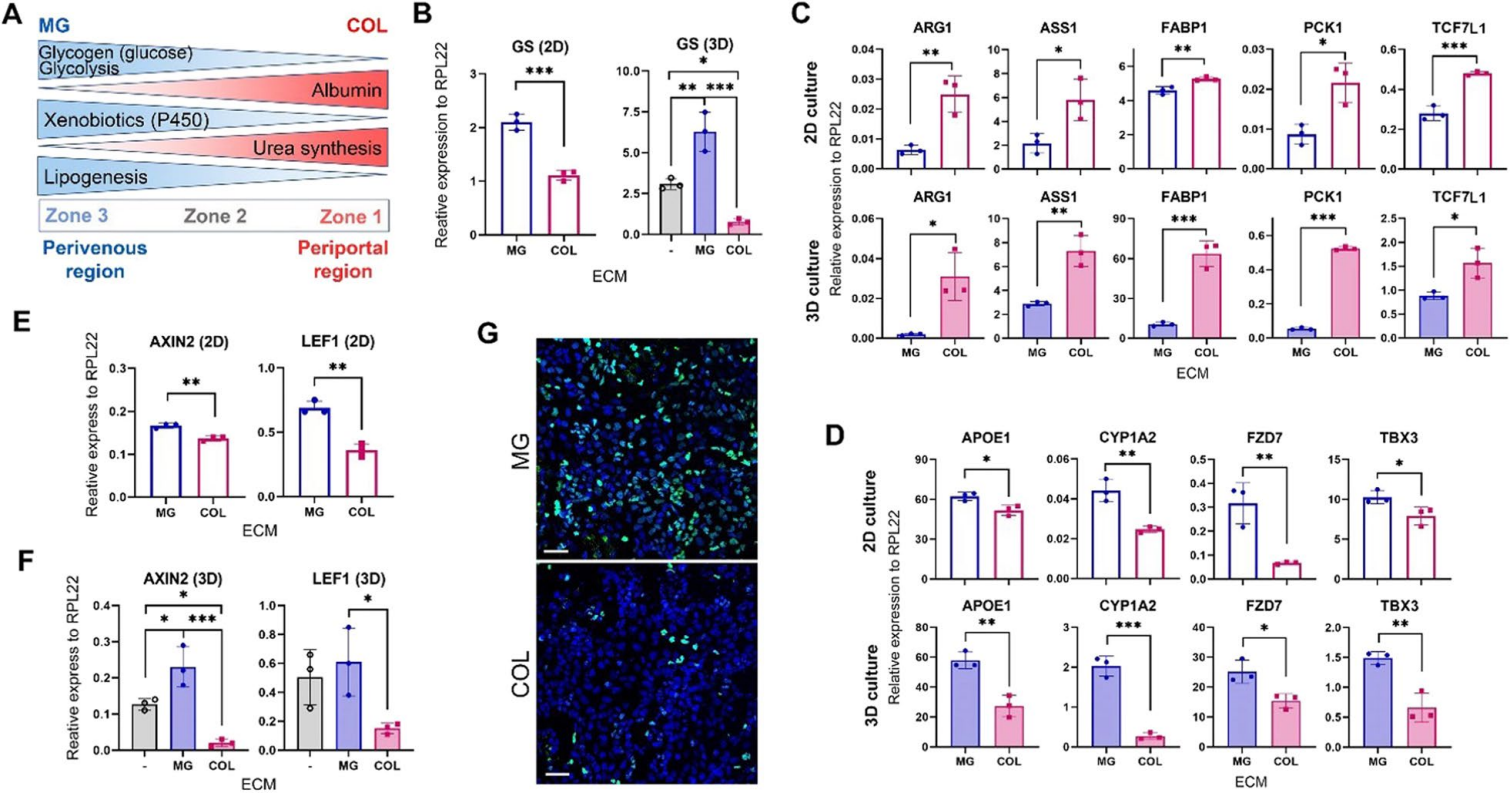
MG and COL modulate the maturation of HLCs with distinct zonation characteristics and Wnt signalling. **A** Schematic summarizes the data of HLCs cultured with MG and COL from previous sections and their association with hepatic zonation. **B** mRNA expression of zone 3 marker glutamine synthetase (GS) in H1 HLCs with MG or COL in both 2D and 3D cultures by RT-qPCR. **C, D** mRNA expression of zone 1 (**C**) and zone 3 (**D**) markers in H1 HLCs with MG or COL in both 2D (upper) and 3D (lower) cultures by RT-qPCR. **E, F** mRNA expression of the Wnt signalling target genes, LEF1 and AXIN2 in HLCs in 2D (**E**) or 3D (**F**) cultures by RT-qPCR. **G** Representative images of immunostaining with LEF1 antibody in HLCs on MG and COL. Scale bar = 50 µm. All PCR data are presented as mean ± SD from three independent differentiation experiments (*n* = 3). *, **, and *** represent *P* < 0.05, 0.005, and 0.0005, respectively, by unpaired two-tailed *t*-test (**B**-2D, **C**–**E**) and one-way ANOVA (**B**-3D, **F**)

Our data above clearly demonstrate that MG and COL differentially regulate the differentiation, maturation, and functionality of the hESC-derived HLCs. COL enhanced ALB production and ureagenesis in HLCs, while MG improved the expression and function of CYP3A as well as promoted glycolysis and glucose–glycogen conversion (Fig. 6A). Altogether, it appears that the differential effects of MG and COL on HLCs correspond well with hepatic zonation in the liver lobules [21, 41, 42]. MG facilitated the differentiation of early-differentiating hepatic cells into HLCs of perivenous zone 3, while COL aided their differentiation into hepatocytes of periportal zone 1 (Fig. 6A). Moreover, glutamine synthetase (GS), a well-established hepatocyte zonation marker with high expression in the perivenous area [42], showed significantly higher levels in the HLCs cultured with MG than with COL (Fig. 6B; Additional file 1: Fig. S6A). To further validate the differential effects of MG and COL on hepatic zonation, more markers reported to be differentially expressed in the zones 1 and 3 were examined by RT-qPCR [42–44]. It showed that COL-HLCs highly expressed zone 1-specific genes including ARG1, ASS1, FABP1, PCK1 and TCF7L1 while MG-HLCs revealed higher levels of zone 3-specific genes such as APOE1, CYP1A2, FZD7 and TBX3 (Fig. 6C and D; Additional file 1: Fig. S6B and C), and in fact, ALB is also a zone 1 marker [42] and showed higher expression in COL cultures (Figs. 1C and E and 2C and D). These data support that MG and COL in HLC cultures affect their differentiation and function to resemble hepatocytes of different hepatic zones.

Furthermore, it has been reported that the Wnt/β-catenin signalling pathway plays a vital role in establishing hepatocyte zonation and is more active in the perivenous area [45–47]. To explore whether Wnt signalling could be different in MG- and COL-cultured HLCs, we verified the expression of direct targets of Wnt—LEF1 and AXIN2 and showed that both were expressed significantly higher in MG-HLCs than in COL-HLCs (Fig. 6E and F; Additional file 1: Fig. S6D and E). Correspondingly, immunostaining of HLCs also showed a more abundant LEF1 protein expression in HLCs from MG cultures than COL (Fig. 6G; Additional file 1: Fig. S6F), supporting that MG and COL may affect hepatocyte zonation through regulating Wnt signalling.

## Discussion

In this study, we investigated the impact of COL and MG on the maturation and function of hESC-derived HLCs using various assays and showed that COL and MG differentially affect the specification and functions of these HLCs. Interestingly, the distinct effects of MG and COL appear to be associated with the zonation in the liver lobules [20, 22, 41], in which COL promotes the hepatic differentiation more towards periportal zone 1 cells, while MG enhances the differentiation and functions more towards perivenous zone 3. Our finding corresponds with the higher expression of COL in the portal stroma [26] and demonstrates that ECM has an important role in regulating the differentiation, maturation, and functionality of hepatocytes from early hepatic progenitors.

Many factors are thought to contribute to the formation of liver lobule zonation, such as nutrients, oxygen, hormones, and morphogens. Among them, nutrients and oxygen are high in periportal zone 1 and form a gradient along the portal–central axis [21, 48, 49]. In our experiments, both MG and COL are applied to parallel HLC cultures with the same source of differentiating cells and culture medium. Therefore, cell autonomous and other environmental factors, except MG and COL, are unlikely to account for the differential zonal functions in these HLCs. Additionally, our results show that HLCs cultured with MG exhibit higher Wnt activity than those cultured with COL, implying that MG and COL may differentially regulate HLCs zonal functions through divergent effects on Wnt signalling. Wnt signalling is crucial for hepatic zonation and is highly active in the perivenous region of the liver lobules [45–47]. One of the main ECM components in MG is laminin, and recombinant laminin LN211E8 has been reported to upregulate Wnt signalling in hPSCs [50]. Thus, high laminin in MG might account for higher Wnt signalling. Although in the liver, laminin is mainly localized in the bile ductules at the portal tracts, the predominant ECM component in the area is collagen [26], which might explain why MG-HLCs display more zone 3 features. However, a previous work reports that purified individual ECMs, including laminin, type I or IV collagen, and fibronectin, have no differential effect on ALB-synthesizing mouse hepatic stem cells [51]. However, the study used no other functional assays except ALB synthesis, making it difficult to compare the two studies.

Furthermore, MG is more complex than a single purified ECM component, containing not only an assortment of ECM proteins but also multiple growth factors [30], which may also affect HLC differentiation and maturation. Although a growth factor-reduced MG was used in our study, we cannot completely exclude the possibility that residue growth factors in combination with other ECM proteins might have resulted in elevated Wnt signalling in HLCs. Moreover, the different isoforms of laminin have been shown to have different effects on hepatocyte differentiation [52]. Therefore, further studies are required to dissect the exact mechanisms by which COL and MG may regulate Wnt signalling and hepatic functional zonation during hepatocyte differentiation, possibly using a defined individual or combination of ECM components.

## Conclusions

Our study provides strong evidence that MG and COL differentially affect HLCs functions, demonstrating the importance of ECM in modulating differentiation and functionality of human hepatocytes derived from hPSCs. These findings will benefit researchers working in the field of hPSCs to derive human hepatocytes for regenerative medicine, disease modelling, and drug discovery. It becomes increasingly evident that a single hepatocyte does not perform all the hepatic functions as the spatial localization in the liver lobules influences their functionality. Application of different ECM components may facilitate the generation of specific groups of hepatocytes for the functions of our interests. In addition, our findings, together with tissue engineering technology, could facilitate the generation of liver tissues that resemble the liver lobular structure by applying different ECMs along the periportal–pericentral axis.

### Supplementary Information

**Additional file 1:** Supplementary Figures and Tables.

## Data Availability

All data and materials except hESCs will be available. The hESCs are under original MTA restrictions.

## Abbreviations

A1AT: Alpha-1 anti-trypsin
AA: Activin A
AFP: α-Fetoprotein
ALB: Albumin
bFGF: Basic fibroblast growth factor
BMP4: Bone morphogenesis protein 4
COL: Type I collagen
CYP450: Cytochrome P450
ECM: Extracellular matrix
FA: Fatty acid
GS: Glutamine synthetase
GK: Glucokinase
GYS2: Glycogen synthase 2
hESCs: Human embryonic stem cells
hPSCs: Human pluripotent stem cells
HLCs: Hepatocyte-like cells
KSR: Knockout serum replacement
LD: Lipid droplet
MG: Growth factor-reduced Matrigel
