# Supplementary material for: Extracellular matrix modulates the spatial hepatic features in hepatocyte-like cells derived from human embryonic stem cells
Source: Stem Cell Res Ther. 2023 Nov 1;14:314. doi: 10.1186/s13287-023-03542-x (PMC10619266; doi:10.1186/s13287-023-03542-x)
Supplement: Supplementary file 1 — Additional file 1: Supplementary Figures and Tables. [file 13287_2023_3542_MOESM1_ESM.pdf]

Supplementary information

Figure S1

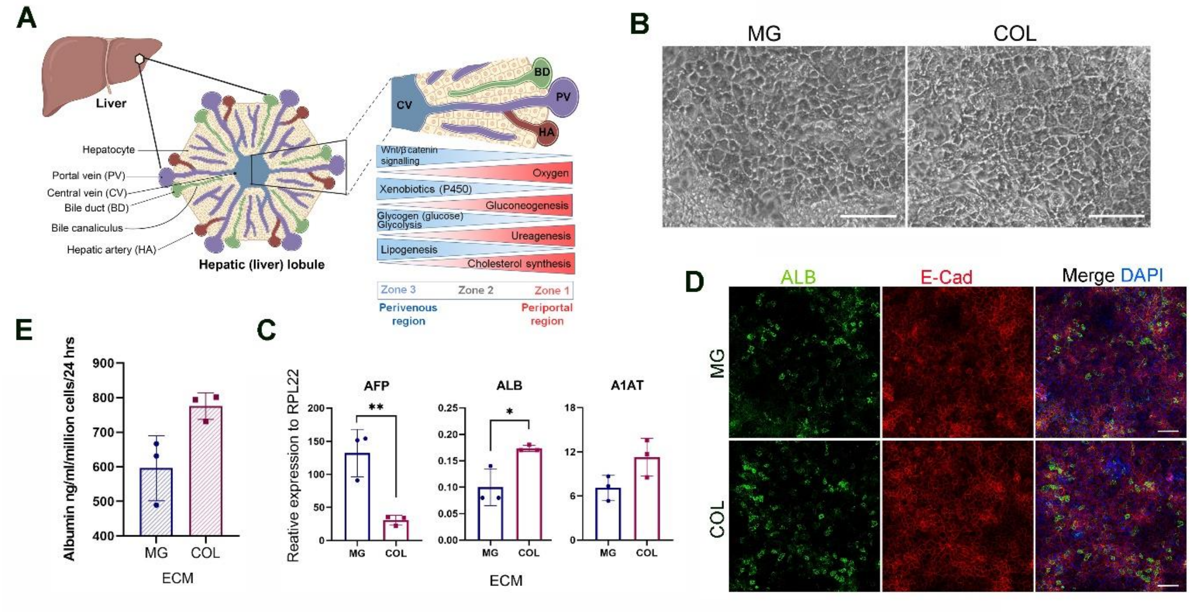

Fig. S1 Supplementary to Fig. 1 with data from H7 hESCs. **A** Illustration depicting the structure of liver lobules. **B** Representative phase-contrast images of HLCs derived from H7 hESCs cultured in Matrigel (MG) and type I collagen (COL). Scale bar = 100 µm. **C** Comparison of indicated mRNA expression in H7 hESC-derived HLCs cultured in MG and COL by RT-qPCR. Data are presented as in Fig. 1C. (n = 3). **D** Immunostaining with indicated antibodies in H7 hESC-derived HLCs cultured on MG and COL. Scale bar = 25 µm. **E** Albumin secretion in H7 hESC-derived HLCs on MG and COL. Data are presented as in Fig. 1F (n = 3).

Figure S2

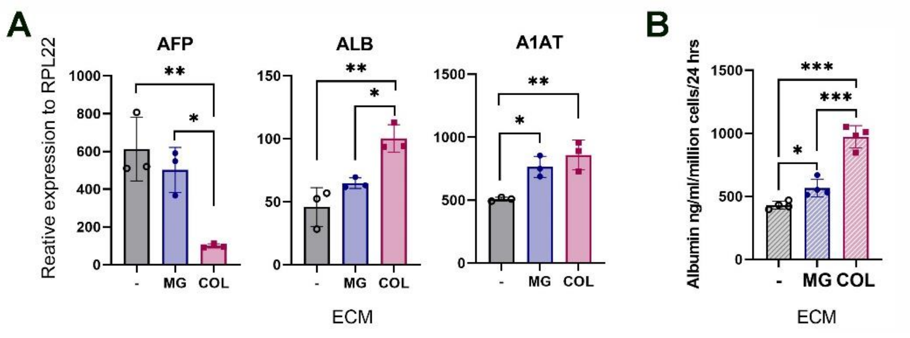

Fig. S2 Supplementary to Fig. 2 with data from H7 hESCs. **A** mRNA expression of indicated genes in 3D cultures with indicated ECM. Data are presented as shown in Fig. 2C (n = 3). (B) Albumin secretion in cells of A. Data are presented as shown in Fig. 2E (n = 4).

Figure S3

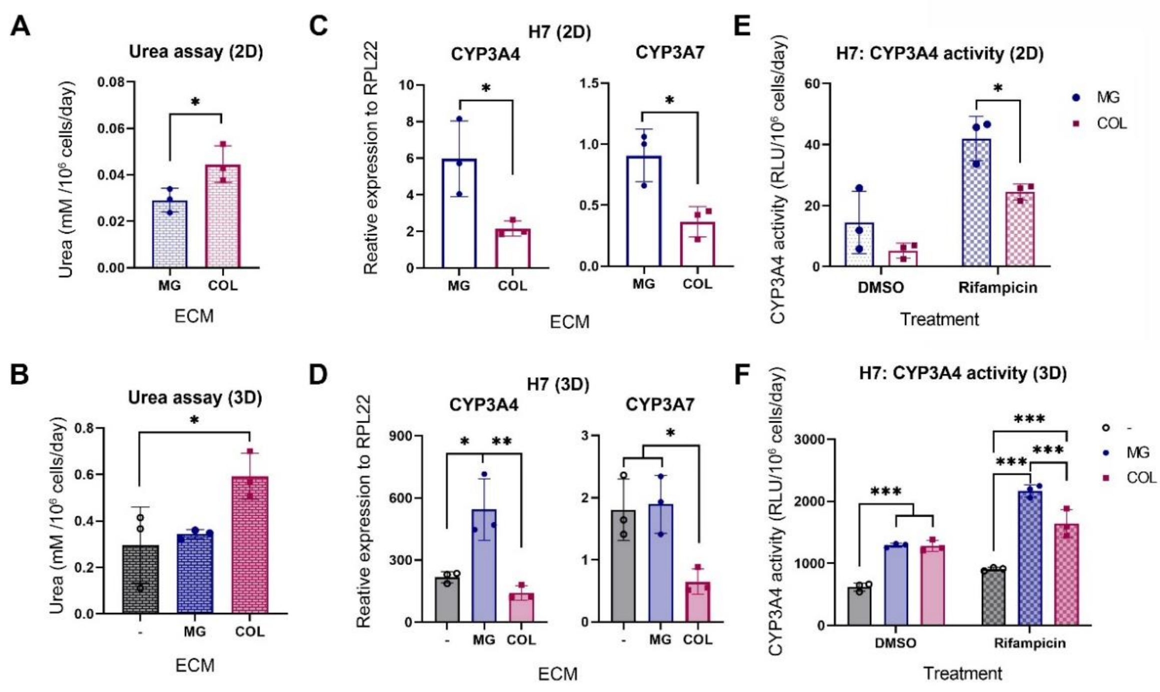

Fig. S3 Supplementary to Fig. 3 with data from H7 hESCs. **A, B** Urea assay in H7 hESC-derived HLCs cultured in 2D (A, n = 3) or 3D (B, n = 3) culture systems with indicated ECM. **C, D** mRNA expression of CYP3A4 and CYP3A7 in H7 hESC-derived HLCs in 2D (C, n = 3) or 3D (D, n = 3) culture systems with indicated ECM by RT-qPCR. **E, F** CYP3A4 activity in H7 hESC-derived HLCs in 2D (E, n = 3) or 3D (F, n = 3) cultures with indicated ECM in the presence or absence of rifampicin stimulation. Data are presented as shown in Fig. 3.

Figure S4

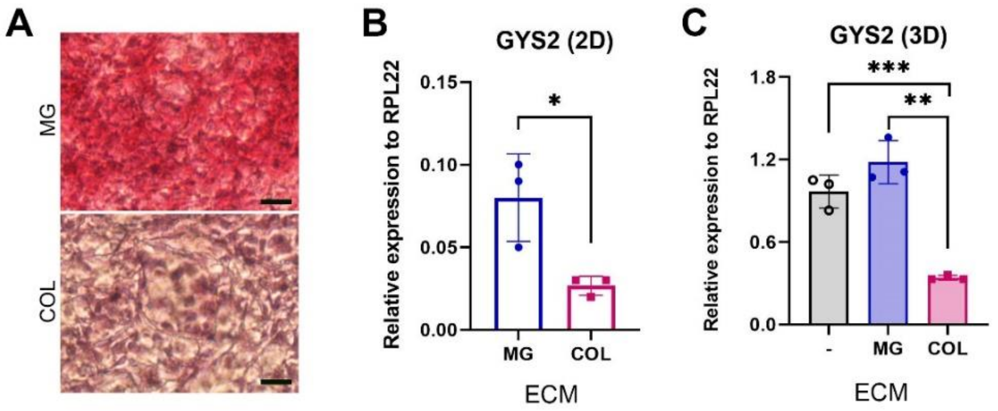

Fig. S4 Supplementary to Fig. 4 with data from H7 hESCs. **A** PAS staining of H7 hESC-derived HLCs cultured on MG and COL. Scale bar = 50 µm. **B, C** RT-qPCR showing GYS2 expression in H7 hESC-derived HLCs with MG and COL in both 2D (B) and 3D (C) culture systems. Data presented as mean ± SD of independent differentiation experiments (n = 3). *, ** and *** are as described in Fig. 4.

Figure S5

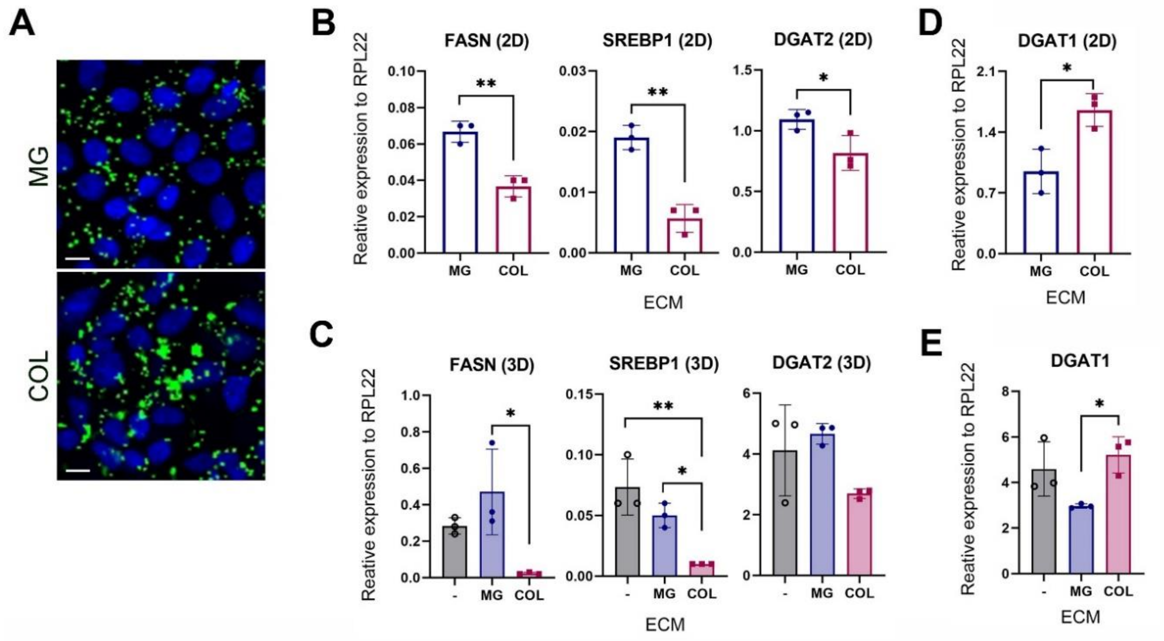

Fig. S5 Supplementary to Fig. 5 with data from H7 hESCs. **A** BODIPY staining of LDs in H7 hESC-derived HLCs cultured on MG or COL. Scale bar = 50 µm. **B-E** Expression of indicated genes in H7 hESC-derived HLCs with indicated ECM in both 2D (B, D) and 3D (C, E) cultures by RT-qPCR. * and ** represent p < 0.05 and 0.005, respectively by either unpaired two-tailed t test (B, D) or one-way ANOVA (C, E) with three independent biological samples for each condition. Data are presented as shown in Fig. 5.

Figure S6

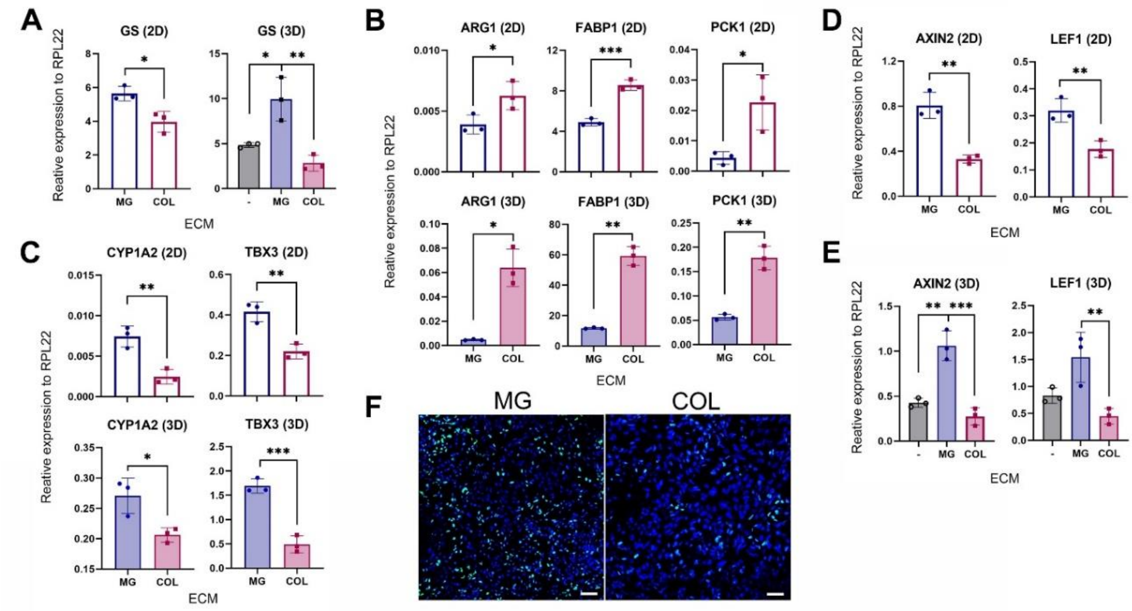

Fig. S6 Complementary to Fig. 6 with data from H7 hESCs. **A** GS expression in H7 hESC-derived HLCs cultured with MG or COL as indicated in both 2D and 3D cultures by RT-qPCR. **B, C** mRNA expression of zone 1 (B) and zone 3 (C) markers in H7 hESC-derived HLCs with MG or COL in both 2D (upper) and 3D (lower) cultures by RT-qPCR. **D, E** Expression of Wnt signalling target genes in H7 hESC-derived HLCs cultured with MG or COL as indicated in 2D (D) and 3D (E) cultures by RT-qPCR. Data presented and analysed as shown in Fig. 6. **F I**mmunostaining of LEF1 in H7 hESC-derived HLCs on MG and COL. Scale bar = 50 μm.

Table S1. Primer sequences used for quantitative RT–PCR

Gene	Forward Primer 5'-3'	Reverse Primer 5'-3'
A1AT	GATCAACGATTACGTGGAGAAGG	CCTAAACGCTTCATCATAGGCA
AFP	TGGGACCCGAACTTTCCA	GGCCACATCCAGGACTAGTTTC
ALB	CCTTTGGCACAATGAAGTGGGTAACC	CAGCAGTCAGCCATTTCACCATAGG
APOE	GGGTCGCTTTTGGGATTACCTG	CAACTCCTTCATGGTCTCGTCC
ARG1	TCATCTGGGTGGATGCTCACAC	GAGAATCCTGGCACATCGGGAA
ASS1	GCTGAAGGAACAAGGCTATGACG	GCCAGATGAACTCCTCCACAAAC
AXIN2	GTGAGGTCCACGGAAACTGT	TGGCTGGTGCAAAGACATAG
CYP1A2	TCATCCTGGAGACCTTCCGACA	GCCACTGGTTTACGAAGACACAG
CYP3A4	AAGTCGCCTCGAAGATACACA	AAGGAGAGAACACTGCTCGTG
CYP3A7	GATCTCATCCCAAACTTGGCCG	CATAGGCTGTTGACAGTCATAAATA
DGAT1	CTCTTCCACTCCTGCCTGAA	AGGTACTCGTGGAAGAAGGC
DGAT2	GTGAGGGCAGTAGTAGGCAT	ATGTCATCAGCCACCCAAGA
FABP1	AGTGGTTCAGTTGGAAGGTGA	GCAGACTTGTTTAAATTCTCTTGC
FASN	AACTTGCAGGAGTTCTGGGAC	TTGGGGTGGACTCCGAAGA
GS	CTGCCATACCAACTTCAGCACC	ATAGGCACGGATGTGGTACTGG
GYS2	TGGTAAATATGTCGTTGCCCAA	GTAGTGTAGCGTGGGTTGTAAAT
LEF1	AATGAGAGCGAATGTCGTTGC	GCTGTCTTTCTTTCCGTGCTA
PCK1	*AAGGTGTTCCCATTGAAGG*	*GAAGTTGTAGCCAAAGAAGG*
SREBP1C	GCTGCTGACCGACATCGAA	ATGTGGCAGGAGGTGGAGAC
TBX3	GGACACTGGAAATGGCCGAAGA	GCTGCTTGTTCACTGGAGGACT
TCF7L1	TCAAGGACACGAGGTCACCATC	GGAGAAGTGGTCATTGCTGTAGG
